# Clinical Features of Severe or Fatal *Mycoplasma pneumoniae* Pneumonia

**DOI:** 10.3389/fmicb.2016.00800

**Published:** 2016-06-01

**Authors:** Koichi Izumikawa

**Affiliations:** Department of Infectious Diseases, Nagasaki University Graduate School of Biomedical SciencesNagasaki, Japan

**Keywords:** *Mycoplasma pneumoniae*, corticosteroids, fulminant pneumonia, hyperimmune response, LDH

## Abstract

*Mycoplasma pneumoniae* is one of the most common causes of community-acquired pneumonia in children and young adults. The incidence of fulminant *M. pneumoniae* pneumonia (MPP) is relatively rare despite the high prevalence of *M. pneumoniae* infection. This literature review highlights the clinical features of fulminant MPP by examining the most recent data in epidemiology, clinical presentation, pathogenesis, and treatment. Fulminant MPP accounts for 0.5–2% of all MPP cases and primarily affects young adults with no underlying disease. Key clinical findings include a cough, fever, and dyspnea along with diffuse abnormal findings in radiological examinations. Levels of inflammatory markers such as white blood cells and C-reactive protein are elevated, as well as levels of lactate dehydrogenase, IL-18, aspartate transaminase, and alanine transaminase. The exact pathogenesis of fulminant MPP remains unclear, but theories include a delayed hypersensitivity reaction to *M. pneumoniae* and the contribution of delayed antibiotic administration to disease progression. Treatment options involve pairing the appropriate anti-mycoplasma agent with a corticosteroid that will downregulate the hypersensitivity response, and mortality rates are quite low in this treatment group. Further research is necessary to determine the exact pathogenesis of severe and fulminant types of MPP.

## Introduction

*Mycoplasma pneumoniae* is a common cause of atypical pneumonia often seen in youths and accounts for 10–15% of cases in Japan (Ishida et al., 2004; Miyashita et al., 2005). *M. pneumoniae* pneumonia (MPP) is typically mild and characterized by a persistent dry cough, and sometimes self-limiting pneumonia cured with no medication, fulminant cases with severe complications such as respiratory failure, hypoxia, and others have been recognized. Key clinical findings of fulminant MPP involve respiratory failure with diffuse consolidation or an abnormal interstitial pattern on a chest radiograph. Material for this review is based on two review articles by Chan and Welsh (1995) and Izumikawa et al. (2014), along with one case series study by Miyashita et al. (2007). **Table 1** indicates the characters of these three articles. Although the definition of “fulminant MPP” has not been established, we defined “fulminant MPP” as confirmed MPP cases with respiratory failure or fatal cases without respiratory failure in this review.

**Table 1:** Two review articles and one case series article.

Reference	Type of article	Number of cases	Unique points
Chan and Welsh, 1995	Review	46	Three category; Non-fatal respiratory failure (*n* = 26), fatal respiratory failure (*n* = 13), and fatal without respiratory failure (*n* = 7)
Miyashita et al., 2007	Case series	13	13 cases with acute respiratory failure (ARF) and 214 cases without ARF
Izumikawa et al., 2014	Review	52	All cases with respiratory failure

## Epidemiology

Approximately 0.5–2% of all MPP cases are the fulminant type, but the exact frequency of fulminant MPP is unclear (Chan and Welsh, 1995; Miyashita et al., 2007). Chan and Welsh (1995) reported that fulminant MPP occurs most frequently in adolescent males, especially in those with a history of smoking. Japanese fulminant MPP cases indicated no such trend (Miyashita et al., 2007; Izumikawa et al., 2014). Our data revealed that almost 50% of the 52 fulminant MPP cases occurred in patients aged 20–49 years, and 13.5% occurred in the elderly (age > 70 years). Only Four cases occurred in younger patients (age < 20 years). Although several cases of fulminant MPP were reported among children (Park et al., 2012), accurate frequency has not been described yet. The actual mortality rate in fulminant MPP cases is also unknown, with Chan and Welsh (1995) reporting 3–5% in 1980s and our review indicating a total of 2 deaths among 52 Japanese fulminant MPP cases, which is relatively lower (Izumikawa et al., 2014). Chan reported seven fatal cases of MPP without respiratory failure and causes of death included pulmonary thromboembolism, myocarditis, pneumonia, cerebritis, and psychosis. Extrapulmonary complications may occur, but there are no recent studies or reviews that address how these complications affected mortality rates.

## Clinical and Laboratory Features

The major clinical manifestations of fulminant MPP include a cough, high fever, and hypoxia along with diffuse abnormal findings on radiologic examination. In our review, all 52 patients presented with fever (>37.0°C) on their first visit to the hospital. A relatively high fever (>38.0°C) was observed in 88.5%. Respiratory symptoms such as a cough and dyspnea were observed upon admission in 97.3 and 83.3% of cases, respectively. The frequency of other upper respiratory symptoms and sputum production was lower in fulminant MPP (26.9% had sputum production; Izumikawa et al., 2014). The average duration from onset of infection to the development of respiratory failure was 11.2 days (range, 5–21 days; Izumikawa et al., 2014). Chan and Welsh (1995) and Miyashita et al. (2007) reported durations of 10–15 and 9.3 days, respectively, from onset to first administration of appropriate anti-mycoplasma agents. Both studies reveal a similar timeframe for developing respiratory failure.

Laboratory findings indicated elevated levels of white blood cells (WBC) and C-reactive protein (CRP; Miyashita et al., 2007; Izumikawa et al., 2014). The majority of severe MPP cases exhibited a moderate inflammation response. Liver dysfunction was common in fulminant MPP and indicated by elevations of alanine transaminase (ALT) and aspartate transaminase (AST). The observed liver dysfunction is not necessarily due to direct invasion of the liver tissue by *M. pneumoniae*, and may result from an indirect immunological response by liver tissue to the pathogen. Total protein (TP) and lactate dehydrogenase (LDH) levels are also elevated in fulminant MPP cases (Miyashita et al., 2007; Izumikawa et al., 2014). However, these abnormal findings were not specific to fulminant MPP cases and were present irrespective of disease severity. However, Miyashita et al. (2007) compared the laboratory findings of severe cases with and without acute respiratory failure (ARF), and found that WBC count and levels of CRP, LDH, AST, and ALT were all significantly higher in the ARF cases. Only TP levels were lower in fulminant MPP cases with ARF compared to cases without ARF.

## Radiological Features

Various radiological findings are observed in fulminant MPP cases. Miyashita et al. (2007) indicated that bilateral infiltrates and pleural effusion commonly present in MPP cases with ARF compared to those without ARF. Izumikawa et al. (2014) observed a diffuse interstitial pattern (e.g., reticular, nodular, linear) in 61.5% of cases. A diffuse alveolar pattern with or without air bronchogram was observed in 25.0% and a mixed interstitial and alveolar pattern was observed in 13.5%. Pleural effusion was noted in 13.5%, and these were categorized as either alveolar or mixed pattern cases. It is unclear what the differences between these radiological findings indicate, and observed findings may change throughout the course of the illness. Interestingly, the radiological findings and severity of inflammation reflected by inflammation markers indicated that mixed pattern cases had higher CRP levels compared to those of interstitial or alveolar pattern cases, though no statistical differences were observed. These findings indicate a possible correlation between inflammatory level and radiological findings. Furthermore, this indicated that radiological findings are closely related to the body’s immunological response to *M. pneumoniae*, and not the local existence of *M. pneumoniae* itself in the affected region of the lung.

## Pathological Features

Few case studies describe the pathological findings found through transbronchial lung biopsy, open lung biopsy, and autopsy from fulminant MPP (Izumikawa et al., 2014). Acute bronchiolitis was identified in the early phase of infection, followed by organizing pneumonia and alveolitis with or without granuloma formation in the recovery phase. These findings, however, were not acquired from one specific case. Among fatal MPP cases, very few cases with diffuse alveolar damage have been reported (Chan and Welsh, 1995; Miyashita et al., 2007; Izumikawa et al., 2014).

## Pathogenesis

The literature details how the host’s cell-mediated immunity plays an important role in the development of typical MPP (Evengard et al., 1994; Chan and Welsh, 1995; Waites and Talkington, 2004). Factors associated with the virulence of *M. pneumoniae* include the following: (i) Toxic byproducts of inflammation can cause cell damage through direct interaction between human host cells (e.g., adherence to bronchoepithelial cells, toxin production, reactive oxygen species, and cytokines). (ii) indirect interaction of immunological or allergic reaction to *M. pneumoniae* infection, and this reaction can influence how the disease progresses (Fernald et al., 1981; Tanaka et al., 2002).

Although the mechanism and etiology of fulminant MPP are largely unknown, three possible hypotheses appeared in the literature: (i) a hyperimmune response that originates in the lung as a result of repeated childhood *M. pneumoniae* infections; (ii) loss of the ability to eradicate *M. pneumoniae* from the lung in primary infection resulting in longer-lasting *M. pneumoniae* infection in the lung, which may cause a hyperimmune response, and (iii) overactive innate immune response such as macrophage activation via heterodimerization of Toll-like receptors two and six of the bronchoepithelial cells to *M. pneumoniae* lipoproteins (Takeuchi et al., 2001). Tanaka et al. (2002) demonstrated that the levels of serum IL-18, but not of interferon, were higher in patients with fulminant MPP compared to those in mild cases, and this was correlated with the number of affected lung lobes. Recently, Miyashita et al. (2015) supported the notion of a positive correlation between IL-18 and LDH levels in severe MPP cases. Thus, an excessive host-cellular response with Th1 cytokines and IL-18 may play a critical role in the development of fulminant status.

## Treatment

All previous reports under review indicated that the delayed use of anti-mycoplasma drugs, such as macrolides (erythromycin, clarithromycin, and azithromycin), tetracyclines, and quinolones contributed to the development of fulminant MPP (Chan and Welsh, 1995; Miyashita et al., 2007; Izumikawa et al., 2014). β-Lactams and aminoglycosides, both of which have no potent activity against *M. pneumoniae* infection, were used as the initial treatment in 61.5% of cases, and rates of both inappropriate and no-treatment cases reached 78.8% (Izumikawa et al., 2014). Corticosteroids are a reasonable treatment option, especially in fulminant MPP cases that present with a hyperactive immune response, given that this class of drugs acts by downregulating the cell-mediated immune response; numerous reports indicate a positive response (Evengard et al., 1994; Chan and Welsh, 1995; Takiguchi et al., 2001; Tsuruta et al., 2002). Currently, there is not enough data available to establish specific pharmacological guidelines to treat fulminant MPP. The literature describes a relatively high dose of methylprednisolone (>500 mg/day) combined with appropriate anti-mycoplasma agents that effectively improved symptoms in the majority of patients within 3–5 days of corticosteroid treatment (Izumikawa et al., 2014). Thirteen cases of severe or refractory MPP cases required mechanical ventilation, and nine of these patients received high dose corticosteroids and anti-mycoplasma agents (Miyashita et al., 2007). Corticosteroid dosage was gradually tapered within a week in almost all cases (Izumikawa et al., 2014). The prolonged or inappropriate usage of corticosteroids may cause excess downregulation of cell-medicated immunity and result in immunosuppression, making the individual more susceptible to a more severe *M. pneumoniae* infection or opportunistic infections such as those by *Pneumocystis, Mycobacterium*, or *Cytomegalovirus* species. Careful consideration is required when determining corticosteroid usage.

**FIGURE 1:**
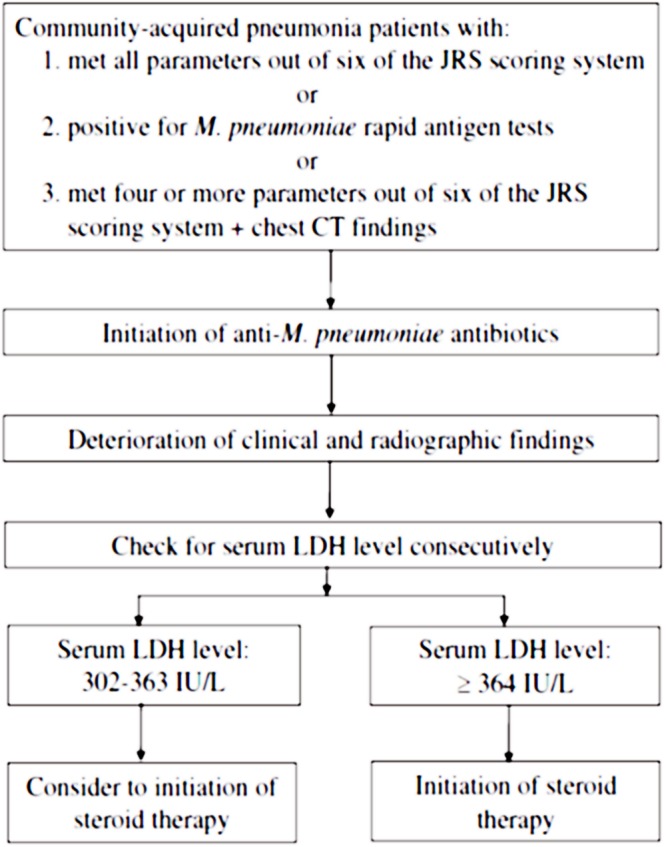
**A stepwise algorithm to correlate diagnosis, serum markers, and initiation of corticosteroid therapy in *Mycoplasma pneumoniae* pneumonia (MPP).** The permission for usage of this figure was acquired from both Dr. Naoyuki Miyashita and the Journal of Infection and Chemotherapy. JRS, Japanese Respiratory Society. *M. pneumoniae* rapid antigen tests; Ribotest Mycoplasma (Asahi kasei Pharma Co., Tokyo, Japan) and PrimeCheck Mycoplasma Antigen (Alfresa Pharma Co., Osaka, Japan).

A recent study recognized that serum LDH levels are significantly higher in cases of severe MPP compared to the control group at the initiation of corticosteroid therapy, and may represent disease severity among adolescents and adults. Lu et al. (2015) also revealed that serum LDH levels can be used as a biomarker to predict refractory MPP in children. A serum LDH level of 302–364 IU/L seems to be an appropriate criterion for the initiation of corticosteroid therapy in severe or refractory MPP among adolescents and adults (Miyashita et al., 2015). **Figure 1** indicates the algorithm of corticosteroid administration in severe and refractory MPP cases (Miyashita et al., 2015). The Japanese Respiratory Society (JRS) scoring system enables to differentiate atypical pneumonia from bacterial pneumonia by positivity of six factors as follows; (i) age < 60 years old, (ii) no or mild co-morbidity, (iii) paroxysmal cough, (iv) poor findings from chest physical examination, (v) no expectoration or no pathogens in rapid diagnostic tests, and (vi) white blood cell count <10,000/mm^3^ (Miyashita et al., 2006). In addition, both of *M. pneumoniae* antigen, Ribotest Mycoplasma (Asahi kasei Pharma Co., Tokyo, Japan) and PrimeCheck Mycoplasma Antigen (Alfresa Pharma Co., Osaka, Japan) were used as rapid antigen tests for diagnosis of MPP (Miyashita et al., 2015).

## Conclusion

*Mycoplasma pneumoniae* pneumonia usually causes a mild illness, and mortality is quite low. However, severe or fulminant cases do occur, and these cases require early administration of corticosteroids, along with administration of appropriate anti-mycoplasma agents. To date, there are no reports of an increase in cases of fulminant MPP during the recent outbreak of macrolide-resistant MPP in Japan, but future research is needed to examine the correlation between severity of *M. pneumoniae* infection and drug resistance, and also to establish clinical guidelines for management of fulminant MPP.

## Author Contributions

The author confirms being the sole contributor of this work and approved it for publication.

## Conflict of Interest Statement

KI received honoraria from MSD K.K, Sumitomo Dainippon Pharma Co., Ltd, Pfizer Japan, Inc. and Taisyo Toyama Co., Ltd.

## References

[B1] Chan E. D. Welsh C. H. (1995). Fulminant *Mycoplasma pneumoniae* pneumonia. *West. J. Med.* 162 133–142. 7725685 PMC1022647

[B2] Evengard B. Sandstedt K. Bolske G. Feinstein R. Riesenfelt-Orn I. Smith C. I. (1994). Intranasal inoculation of *Mycoplasma pulmonis* in mice with severe combined immunodeficiency (SCID) causes a wasting disease with grave arthritis. *Clin. Exp. Immunol.* 98 388–394. 10.1111/j.1365-2249.1994.tb05502.x 7994902 PMC1534506

[B3] Fernald G. W. Clyde J. W. A. Denny F. W. (1981). *Immunology of Mycoplasma Infection.* New York, NY: Plenum Medical Book Co.

[B4] Ishida T. Hashimoto T. Arita M. Tojo Y. Tachibana H. Jinnai M. (2004). A 3-year prospective study of a urinary antigen-detection test for *Streptococcus pneumoniae* in community-acquired pneumonia: utility and clinical impact on the reported etiology. *J. Infect. Chemother.* 10 359–363. 10.1007/s10156-004-0351-1 15614462

[B5] Izumikawa K. Izumikawa K. Takazono T. Kosai K. Morinaga Y. Nakamura S. (2014). Clinical features, risk factors and treatment of fulminant *Mycoplasma pneumoniae* pneumonia: a review of the Japanese literature. *J. Infect. Chemother.* 20 181–185. 10.1016/j.jiac.2013.09.009 24462437

[B6] Lu A. Wang C. Zhang X. Wang L. Qian L. (2015). Lactate dehydrogenase as a biomarker for prediction of refractory *Mycoplasma pneumoniae* pneumonia in children. *Respir. Care* 60 1469–1475. 10.4187/respcare.03920 26060318

[B7] Miyashita N. Fukano H. Mouri K. Fukuda M. Yoshida K. Kobashi Y. (2005). Community-acquired pneumonia in Japan: a prospective ambulatory and hospitalized patient study. *J. Med. Microbiol.* 54 395–400. 10.1099/jmm.0.45920-0 15770027

[B8] Miyashita N. Kawai Y. Inamura N. Tanaka T. Akaike H. Teranishi H. (2015). Setting a standard for the initiation of steroid therapy in refractory or severe *Mycoplasma pneumoniae* pneumonia in adolescents and adults. *J. Infect. Chemother.* 21 153–160. 10.1016/j.jiac.2014.10.008 25533771

[B9] Miyashita N. Matsushima T. Oka M. Japanese Respiratory S. (2006). The JRS guidelines for the management of community-acquired pneumonia in adults: an update and new recommendations. *Intern. Med.* 45 419–428. 10.2169/internalmedicine.45.1691 16679695

[B10] Miyashita N. Obase Y. Ouchi K. Kawasaki K. Kawai Y. Kobashi Y. (2007). Clinical features of severe *Mycoplasma pneumoniae* pneumonia in adults admitted to an intensive care unit. *J. Med. Microbiol.* 56 1625–1629. 10.1099/jmm.0.47119-0 18033831

[B11] Park S. J. Pai K. S. Kim A. R. Lee J. H. Shin J. I. Lee S. Y. (2012). Fulminant and fatal multiple organ failure in a 12-year-old boy with *Mycoplasma pneumoniae* infection. *Allergy Asthma Immunol. Res.* 4 55–57. 10.4168/aair.2012.4.1.55 22211173 PMC3242064

[B12] Takeuchi O. Kawai T. Muhlradt P. F. Morr M. Radolf J. D. Zychlinsky A. (2001). Discrimination of bacterial lipoproteins by Toll-like receptor 6. *Int. Immunol.* 13 933–940. 10.1093/intimm/13.7.933 11431423

[B13] Takiguchi Y. Shikama N. Aotsuka N. Koseki H. Terano T. Hirai A. (2001). Fulminant *Mycoplasma pneumoniae* pneumonia. *Intern. Med.* 40 345–348. 10.2169/internalmedicine.40.345 11334397

[B14] Tanaka H. Narita M. Teramoto S. Saikai T. Oashi K. Igarashi T. (2002). Role of interleukin-18 and T-helper type 1 cytokines in the development of *Mycoplasma pneumoniae* pneumonia in adults. *Chest* 121 1493–1497. 10.1378/chest.121.5.1493 12006434

[B15] Tsuruta R. Kawamura Y. Inoue T. Kasaoka S. Sadamitsu D. Maekawa T. (2002). Corticosteroid therapy for hemolytic anemia and respiratory failure due to *Mycoplasma pneumoniae* pneumonia. *Intern. Med.* 41 229–232. 10.2169/internalmedicine.41.229 11929187

[B16] Waites K. B. Talkington D. F. (2004). *Mycoplasma pneumoniae* and its role as a human pathogen. *Clin. Microbiol. Rev.* 17 697–728. 10.1128/CMR.17.4.697-728.2004 15489344 PMC523564

